# Comparison of Up-Front Minimally Invasive Esophagectomy versus Open Esophagectomy on Quality of Life for Esophageal Squamous Cell Cancer

**DOI:** 10.3390/curroncol28010068

**Published:** 2021-01-25

**Authors:** Zhenhua Li, Jingge Cheng, Yuefeng Zhang, Shiwang Wen, Huilai LV, Yanzhao Xu, Yonggang Zhu, Zhen Zhang, Donghui Mu, Ziqiang Tian

**Affiliations:** 1 Department of Thoracic Surgery, The Fourth Hospital of Hebei Medical University, Shijiazhuang 050011, China; sky_sea1226@163.com (Z.L.); yuefengzheng1234@163.com (Y.Z.); shiwangwen@163.com (S.W.); huilailv123@163.com (H.L.); yanzhaoxu1234@163.com (Y.X.); yonggangzhu123@163.com (Y.Z.); zhenzhang4321@163.com (Z.Z.); donghuimu123@163.com (D.M.); 2 Department of Orthopedics, The Fourth Hospital of Hebei Medical University, Shijiazhuang 050011, China; jinggecheng@163.com

**Keywords:** esophageal cancer, minimally invasive esophagectomy, open esophagectomy, quality of life, KPS, QLQC-30, OES-18

## Abstract

This study investigates whether minimally invasive esophagectomy (MIE) is a safe and effective way for patients with resectable esophageal cancer by comparing the short-term quality of life (QOL) after minimally invasive esophagectomy and open esophagectomy (OE). A total number of 104 patients who underwent esophagectomy from January 2013 to March 2014 were enrolled in this study. These patients were divided into two groups (MIE and OE group). Three scoring scales of quality of life were used to evaluate QOL before the operation and at the first, third, sixth and twelfth months after MIE or OE, which consist of Karnofshy performance scale (KPS), the European Organization for Research and Treatment questionnaire QLQC-30 (EORTC QLQC-30) and esophageal cancer supplement scale (OES-18). The MIE group was higher than the OE group in one-year survival rate (92.54% vs. 72.00%). Significant differences between the two groups were observed in intraoperative bleeding volume (158.53 ± 91.07 mL vs. 228.97 ± 109.33 mL, *p* = 0.001), and the incidence of postoperative pneumonia (33.33% vs. 58.62%, *p* = 0.018). The KPS of MIE group was significantly higher than the OE group at the first (80 vs. 70, *p* = 0.004 < 0.05), third (90 vs. 80, *p* = 0.006 < 0.05), sixth (90 vs. 80, *p* = 0.007 < 0.05) and twelfth months (90 vs. 80, *p* = 0.004 < 0.05) after surgery. The QLQC-30 score of MIE group was better than OE group at first and twelfth months after the operation. The OES-18 score of MIE group was significantly better than OE group at first, sixth and twelfth months after surgery. The short-term quality of life in MIE group was better than OE group.

## 1. Introduction

Esophageal cancer was a common type of malignant tumor of the digestive tract, and the incidence rate has increased dramatically over the past years [1]. It was one of the leading causes for cancer-associated mortality in China, and the incidence and mortality rate of esophageal cancer were ranked 4th in 2015 [2]. The treatment therapies for esophageal cancer include surgery, radiotherapy and chemotherapy, and surgery was regarded as the best option for patients with resectable esophageal cancer [3,4,5]. Open esophagectomy was the traditional strategy for treating esophageal cancer, including Sweet surgery (left thoracic, one incision) and Ivor-Lewis surgery (right chest posterolateral and upper abdomen median, two incisions). It was reported that the R0 resection rate of Ivor Lewis surgery was higher than Sweet surgery (86.2% vs. 73.2%), and the amount of lymph nodes dissected by Ivor Lewis surgery was significantly larger than Sweet surgery (27.0 ± 12.4 vs. 17.0 ± 6.4), and the five year survival rate of Ivor Lewis surgery was higher than Sweet surgery (35% vs. 19%) [6]. Minimally invasive esophagectomy (MIE) has been proven to be beneficial for esophagus cancer, on account of decreasing the incidence of complications and similar curative effect compared with open esophagectomy [7]. Nowadays, postoperative quality of life (QOL) has been a concern for an increasing number of surgeons and patients except survival rates [8].

Quality of life was a broad and multidimensional construct, including various domains of physical, psychological and social health. The standard questionnaires which used to assess the quality of life in esophageal cancer patients were the European organization for research and treatment of cancer Quality of Life Questionnaire-Core 30 (EORTC QLQ-C30) and esophageal cancer supplement scale (OES-18). A meta-analysis showed that the postoperative symptoms of constipation, speech problems and insomnia increased for a long time after MIE [9]. The use of MIE was associated with modestly improved perioperative outcomes comparing with OE in western esophageal cancer patients [10]. However, few studies addressed the quality of life in esophageal cancer patients after the procedure in Chinese esophageal cancer patients. In our study, we compared the quality of life of Chinese esophageal cancer patients after minimally invasive esophagectomy and open esophagectomy by performing Karnofshy performance scale (KPS), EORTC QLQC-30 and OES-18, to further investigate the safety and effectiveness of minimally invasive esophagectomy.

## 2. Materials and Methods

### 2.1. Patients

A total of 104 Chinese patients with esophageal cancer were enrolled from January 2013 to March 2014 in Fourth Hospital of Hebei Medical University Hospital. Basic and clinicopathological information from all patients were collected. Based on the operative method, patients were divided into two groups, minimally invasive esophagectomy (MIE) group and open esophagectomy (OE) group. All of the patients underwent gastroscopy, and the tumors needed to be confirmed as esophageal squamous cell carcinoma by histological results before surgery. Patients in both groups would undergo chest CT scan, abdominal CT scan, electrocardiogram, pulmonary and cardiac function testing, head CT scan, cervical spine CT scan, bronchoscope, ultrasound gastroscopy and PET/CT if necessary. The following exclusion criteria were applied to all patients: patients with distant metastases, patients with operational contraindications, and patients who received radiotherapy or chemotherapy beforehand. Operational contraindications included (1) patients with severe cardiopulmonary insufficiency or serious diseases who could not tolerate surgery; (2) patients with tumor invaded surrounding important tissues and organs shown by preoperative imaging examinations that could not be removed by surgery; (3) patients with distant metastases shown by preoperative imaging examinations, such as hepatic metastases, pulmonary metastasis and bone metastases; (4) patients first diagnosed with esophageal small cell carcinoma; and (5) patients who underwent chest or abdominal surgery in the past who could not undergo surgery again.

### 2.2. Quality of Life Measurements

The quality of life measurements was performed by the European Organization for Research and Treatment of Cancer Quality of Life Core Questionnaire 30 (EORTC QLQ-C30) and the esophageal cancer supplement scale (OES-18). The QLQ-C30 questionnaire included a global health and quality-of-life scale, 5 functional scales (physical, role, cognitive, emotional and social), 3 symptom scales (fatigue, pain and nausea and vomiting) and 6 single-item symptom measures (insomnia, dyspnea, constipation, diarrhea, decreased appetite and economic difficulties). The OES 18 included 4 symptom scales (dysphagia, troublesome eating, gastroesophageal reflux and pain) and 6 single-item scales (troublesome saliva swallowing, choking, having a dry mouth, troublesome taste, troublesome coughing and troublesome talking). Raw scores from the EORTC QLQ-C30 and OES 18 were transformed according to standard methods [11,12]. For functional scales, a higher score indicated better function, and for symptom and single-item scales, a higher score meant worse or more symptoms. The Karnofsky Performance Scale was an assessment tool for functional impairment, and its ranking ran from 100 to 0, where 100 was “perfect” health and 0 was death; a higher score equaled to better healthy status of patients.

### 2.3. Follow-Up

All patients were followed-up at the first, third, sixth and twelfth months after surgery. All functional and quality of life assessments were performed prior to treatment and at 1, 3, 6 and 12 months after treatment.

### 2.4. Statistical Analysis

Continuous variables were presented as medians and interquartile ranges (IQR), applying *t*-test for data meeting normal distribution and homogeneity and Wilcoxon test for data not meeting normal distribution and homogeneity. Categorical variables were presented as percentages, using Chi-square test for comparison of their difference. A *p* value of lesser than 0.05 was considered statistically significant. These statistical analyses were performed by SPSS version 26.0 (IBM, Chicago, IL, USA).

## 3. Results

### 3.1. Baseline Measurements

In this study, we enrolled 104 esophageal cancer patients (Table 1). These patients were divided into two groups based on the operative method, MIE group and OE group. There were 75 patients in MIE group, and 8 of them were lost to follow-up. A total of 5 patients died in one year after surgery, and one of them died in perioperative period (due to interstitial pneumonia). There were 29 patients in OE group, and 4 of them was lost to follow-up. Seven of them died in one year after surgery, and one of them died in perioperative period (due to empyema). The one-year survival rate of MIE group (92.54%) was significantly higher than OE group (72.00%) (*p* < 0.05). There were no significant differences of gender, age, stage, operation duration, number of dissected lymph nodes, postoperative ventilator support rate and anastomotic leakage rate between the two groups. Both intraoperative bleeding volume and incidence of pneumonia of MIE group were significantly lower than OE group (Table 1).

### 3.2. Quality of Life Evaluation

#### Karnofsky Performance Scale

The median of preoperative KPS scores were the same in the two groups (90 of MIE group, 90 of OE group, *p* = 0.552). The median of postoperative KPS scores of MIE group were significantly higher than OE group at the first, third, sixth and twelfth months (Table 2).

### 3.3. EORTC QLQC-30

EORTC QLQC-30 was also performed to evaluate quality of life of Chinese esophageal cancer patients in our study (Table 3, Figure S1). There were no significant differences of preoperative EORTC QLQC-30 scores between the two groups. At one month after surgery, there were several scales which were better in MIE group, including global health status, role function, physical function, social function, fatigue, pain, dyspnea and economic difficulties. When comparing between groups, no significant changes were present in other scales at this time point. Most scales better in MIE group at one month after operation were still better in MIE group at three months, except economic difficulties. Decreased appetite in MIE group was also better than OE group. Six months later, global health status, role function, social function, fatigue, pain and economic difficulties in MIE group were better than OE group. At twelfth months, there were only three scales better in MIE group, global health status, role function and pain. Other postoperative EORTC QLQC-30 scales did not change between the two groups an any time point.

### 3.4. OES-18

OES-18 was an esophageal cancer-specific module and important supplemental questionnaire of QLQC-30. There were no significant differences of preoperative OES-18 scores between the two groups (Table 4, Figure S2). At one month after operation, the scales which were significantly better in MIE group included dysphagia, troublesome eating, pain, choking, troublesome tasting and troublesome coughing. There were no significant changes presented in other scales at this time point. Except for the 7 scales which were better in MIE group at one month after operation, there was another scale better in MIE group at three months with statistical significance which was having a dry mouth. Six months later, only four scales changed between the two groups significantly, including troublesome eating, pain, choking and troublesome tasting. Moreover, there were only 3 scales better in MIE group, which were troublesome eating, pain and troublesome tasting.

## 4. Discussion

Worldwide, China has a high incidence of esophageal cancer. In the treatment of esophageal cancer, esophageal resection was one of the most effective therapies; however, prognosis of esophageal cancer was still not very good even though diagnosis and operation have been improved in recent years [13], and the 5-year survival rate of advanced esophageal cancer patients was still less than 20% [14]. Nowadays postoperative quality of life (QOL) has been a concern for an increasing number of surgeons and patients [8]. In 1992, Cuschieri reported that endoscope was used to treat esophageal cancer for the first time [15]. Many studies showed that there was obvious advantage of thoracoscopy radical esophagectomy compared with traditional open esophagectomy.

It was reported that one-year survival rate of patients underwent thoracoscopy radical esophagectomy was similar with who underwent open esophagectomy, and there were obvious advantages of thoracoscopy esophagectomy on lymph node dissection based on the better dissection range of lymph nodes [16]. Thoracoscope could show the fine structures of nerve, blood vessel and fascia based on the magnifying effect, and especially good for dissection of vagina vasorum and recurrent laryngeal nerve lymph nodes. In our study, the dissection number of lymph nodes of MIE was similar with OE, but lower than the number previously reported [17]. Mariette et al reported that it was limited that using the dissection number of lymph nodes to evaluate the prognosis, and lymph node metastasis was a better predicter of prognosis than the dissection number of lymph nodes [18]. Consistent with previous studies [19,20], we reported that the intraoperative bleeding volume of MIE group was significantly lower than OE group, because of the clearer operative vision on vascular anatomy due to the magnifying effect thoracoscope. The incidence of postoperative pneumonia in MIE group patients was lower than OE group in both our study and previous studies [19,20], due to the use of single-lumen endotracheal intubation which could decrease lung edema induced by prolonged one-lung ventilation with positive end-expiratory pressure [21,22,23]. Otherwise, the less pain and less effect on chest wall and diaphragm in MIE group could alleviate symptoms of postoperative pneumonia by the less effect on cough and expectoration after MIE.

KPS score was mainly used to evaluate behavioral capability of the patients, and a higher score represented a better healthy status on behavioral capability and a better endurance on the side effect causing by treatment. On the contrary, a lower score meant a worse healthy status, and when the score was lower than 60, the patients could not receive the adjuvant therapy after surgery, such as radiotherapy and chemotherapy. In our study, we found that the KPS score in MIE group was significantly higher than OE group, because of the less effect of MIE on behavioral capability, accounting for the smaller incision in the chest and abdomen, which could cause less pain in the patients.

In our study, the results of EORTC QLQC-30 showed that 3 months later the physical, cognitive, emotional and social function of MIE group were better than OE group, and fatigue, pain, dyspnea and decreased appetite of MIE group were worse than OE group. One year later, role and pain in MIE group were better than in OE group. It was suggested that the quality of life of MIE group patients was better than OE group which was consist with previous study in western patients [24,25]. The scores of pain in both groups were the lowest at sixth month after surgery, and they declared that the effect of surgery on pain gradually decreased in six months. In some patients who exhibited recurrence and metastasis, the score of pain increased from the sixth month to the twelfth month. Therefore, recurrence and metastasis were the main cause of pain instead of surgery six months later.

In esophagus cancer, OES-18 was an important supplement for QLQC-30 based on the impeccable evaluation on symptoms of esophagus cancer patients. According to OES-18, the score of troublesome feeding, pain and troublesome taste of MIE group were lower than OE group, which meant the quality of life was better in MIE group. Recurrent laryngeal nerve lymph nodes dissection was very crucial in three-field lymph node dissection for esophagectomy, which could cause troublesome talking in esophagus cancer patients. Shiozaki et al reported that the rate of recurrent laryngeal nerve lymph node metastasis in patients with esophageal squamous cell carcinoma was 39.16%, and the effect of surgery on talking could last 3 to 6 months [26]. In our study, troublesome talking of MIE group was better than OE group based on this questionnaire at the first and third months after surgery, and there was no difference between the two group at sixth and twelfth month. The questionnaire was design for European people who have differences in ethnicity or territory with Chinese people [27]. Therefore, we needed to design a questionnaire suitable for Chinese esophagus cancer patients.

There were some limitations in our study. First, the follow-up time in our study was limited and a longer-time follow-up would help us to study long-time quality of life of esophagus cancer patients after surgery. Second, the three questionnaires we used in our study had some problems, for example, they were hard to finish for uneducated patients, and some indexes needed to be added into the questionnaire, such as body weight and anemia, which could evaluate general physical status of patients. Third, the effect of adjuvant therapy was not included in our study. In the follow up, we avoided doing it at the first week after radiotherapy or chemotherapy. However, adjuvant therapy did affect quality of life of cancer patients for a short time, and we should evaluate quality of life of Chinese esophagus cancer patients treated with MIE or OE combined with adjuvant therapy in the future.

In our study, we found that MIE had a better effect on quality of life of Chinese esophagus cancer patients. Our study was a retrospective study, and a large-sample, prospective study needs to be performed in the future to confirm these results.

## Figures and Tables

**Table 1:** Basic clinical characteristics of patients.

	MIE Group	OE Group	*p* Value
Number	75	29	
Age (years)	59.68 ± 9.25	61.17 ± 5.71	0.387
Gender (Male/Female)	46/29	18/11	0.945
Location of Tumors (n)			0.981
Upper Thoracic Segment	40	16	
Middle Thoracic Segment	21	8	
Lower Thoracic Segment	14	5	
Stage (n)			0.085
0	1	0	
Ⅰ	31	4	
Ⅱ	29	14	
Ⅲ	13	9	
Ⅳ	1	2	
Operation Duration (min)	290.60 ± 48.33	327.93 ± 98.93	0.072
Dissected Lymph Nodes	10.75 ± 5.98	11.31 ± 6.70	0.980
Intraoperative Bleeding Volume (mL)	158.53 ± 91.07	228.97 ± 109.33	0.001
Postoperative Pneumonia (n)	25	17	0.018
Postoperative Ventilator Support (n)	11	5	0.981
Anastomotic Leakage (n)	11	4	0.909

**Table 2:** The Comparison of Karnofshy performance scale (KPS) scores between the two groups.

KPS	MIE Group	OE Group	*p* Value
Pre-operation	90	90	0.522
1st Month	80	70	0.004
3rd Month	90	80	0.006
6th Month	90	80	0.007
12th Month	90	80	0.004

**Table 3:** The Comparison of QLQC-30 between the two groups.

	MIE Group					OE Group				
	Pre-Operation	1st Month	3rd Month	6th Month	12th Month	Pre-Operation	1st Month	3rd Month	6th Month	12th Month
Global Health Status	90.2 ± 10.56	63.66 ± 14.28	73.0 ± 14.46	79.3 ± 16.50	79.6 ± 27.20	89.89 ± 9.86	47.73 ± 1.91	57.20 ± 8.98	65.5 ± 12.68	62.0 ± 28.93
Function										
Physical	94.7 ± 12.36	55.08 ± 22.41	78.3 ± 25.38	89.1 ± 27.71	88.5 ± 29.54	94.1 ± 10.86	12.7 ± 11.62	50.9 ± 18.23	86.3 ± 24.21	79.0 ± 40.77
Role	89.96 ± 9.47	35.25 ± 29.36	80.3 ± 33.26	89.3 ± 29.03	86.8 ± 34.04	90.2 ± 11.24	9.09 ± 19.74	25.0 ± 29.88	68.1 ± 36.34	67.5 ± 46.67
Cognitive	98.56 ± 4.86	89.62 ± 17.26	90.0 ± 15.32	95.9 ± 14.80	95.0 ± 16.76	97.9 ± 10.25	89.3 ± 12.11	92.42 ± 9.93	94.7 ± 13.00	90.8 ± 21.95
Social	90.1 ± 12.34	66.94 ± 13.43	87.4 ± 15.71	89.3 ± 28.22	84.1 ± 34.75	89.86 ± 7.56	46.2 ± 11.42	69.91 ± 9.59	82.5 ± 14.97	84.1 ± 32.66
Symptom										
Fatigue	2.35 ± 4.86	22.22 ± 17.21	10.5 ± 13.06	6.37 ± 11.38	6.38 ± 16.03	2.11 ± 8.23	35.8 ± 17.12	21.7 ± 11.62	11.6 ± 14.34	15.5 ± 28.02
Nausea and Vomiting	3.55 ± 8.56	6.01 ± 14.92	5.19 ± 12.37	4.92 ± 11.52	3.55 ± 9.68	3.12 ± 10.46	9.85 ± 15.13	5.30 ± 9.47	4.04 ± 8.60	2.50 ± 11.18
Pain	1.22 ± 10.12	6.56 ± 8.21	4.92 ± 8.25	2.46 ± 9.54	4.10 ± 15.71	1.56 ± 9.89	39.8 ± 14.64	24.24 ± 9.93	14.4 ± 11.84	16.6 ± 32.89
Single-item										
Dyspnea	2.33 ± 9.54	20.22 ± 26.02	10.9 ± 16.91	8.74 ± 15.99	7.65 ± 20.54	2.21 ± 10.21	36.36 ± 9.81	22.7 ± 15.89	16.6 ± 22.42	15.0 ± 31.48
Insomnia	3.66 ± 8.23	10.93 ± 24.87	7.10 ± 18.37	6.56 ± 15.89	7.10 ± 15.05	3.01 ± 10.24	3.03 ± 9.81	1.52 ± 7.11	3.03 ± 9.81	15.0 ± 27.52
Decreased Appetite	3.11 ± 5.56	3.28 ± 11.71	1.09 ± 5.98	1.64 ± 9.48	3.83 ± 13.74	2.98 ± 9.21	9.09 ± 18.35	7.58 ± 14.30	3.03 ± 9.81	8.33 ± 21.29
Constipation	3.36 ± 7.23	6.01 ± 19.73	4.92 ± 15.91	4.37 ± 14.24	3.82 ± 12.32	3.12 ± 8.13	12.1 ± 21.93	7.58 ± 14.30	4.55 ± 11.71	8.89 ± 18.24
Diarrhea	4.22 ± 5.63	10.93 ± 23.35	7.65 ± 17.63	6.56 ± 15.89	5.46 ± 13.85	4.89 ± 10.22	12.1 ± 19.37	9.09 ± 15.19	7.58 ± 14.30	5.00 ± 12.21
Economic Difficulties	3.88 ± 10.22	4.37 ± 11.34	3.82 ± 10.71	2.73 ± 11.05	4.37 ± 17.72	4.11 ± 8.65	13.6 ± 22.20	9.09 ± 18.35	7.58 ± 14.30	8.33 ± 21.29
Troublesome Talking	0.78 ± 3.64	20.77 ± 30.53	9.29 ± 15.07	1.09 ± 5.98	0.55 ± 4.27	0.55 ± 4.65	18.1 ± 28.60	18.1 ± 24.62	4.55 ± 11.71	1.67 ± 7.45

**Table 4:** The comparison of OES18 between the two groups.

	MIE Group					OE Group				
	Pre-Operation	1st Month	3rd Month	6th Month	12th Month	Pre-Operation	1st Month	3rd Month	6th Month	12th Month
Dysphagia	10.4 ± 10.89	17.30 ± 15.65	6.19 ± 15.91	2.19 ± 10.89	2.55 ± 10.81	11.2 ± 11.23	22.22 ± 9.07	8.08 ± 7.80	1.52 ± 5.19	3.89 ± 12.10
Troublesome Eating	14.33 ± 8.84	16.12 ± 10.96	8.88 ± 7.74	3.96 ± 8.82	2.32 ± 10.77	15.01 ± 9.47	23.86 ± 8.64	13.64 ± 6.58	6.44 ± 4.40	7.50 ± 14.28
Gastroesophageal Reflux	3.21 ± 7.88	21.86 ± 22.88	13.5 ± 17.36	8.20 ± 11.64	4.37 ± 8.55	3.02 ± 9.51	29.5 ± 18.50	18.9 ± 12.90	9.09 ± 9.93	3.33 ± 6.84
Pain	1.96 ± 10.87	6.37 ± 9.40	2.91 ± 7.00	2.00 ± 7.46	2.91 ± 12.49	2.03 ± 11.46	31.82 ± 7.11	21.72 ± 8.02	14.14 ± 9.19	13.8 ± 16.47
Troublesome Saliva Swallowing	2.23 ± 10.21	3.83 ± 13.74	1.64 ± 9.48	0.55 ± 4.27	2.78 ± 11.13	2.46 ± 11.27	6.06 ± 16.70	3.03 ± 9.81	1.52 ± 7.11	3.33 ± 10.26
Choking	15.5 ± 10.78	18.03 ± 22.42	6.56 ± 15.89	4.37 ± 12.87	2.19 ± 10.31	16.1 ± 11.65	40.9 ± 20.40	25.7 ± 14.30	15.1 ± 16.99	5.00 ± 16.31
Having a Dry Mouth	3.39 ± 7.56	15.85 ± 28.94	9.84 ± 19.57	8.20 ± 16.84	5.46 ± 12.44	3.34 ± 10.25	22.7 ± 26.00	21.2 ± 21.93	10.6 ± 18.93	5.00 ± 12.21
Troublesome Taste	1.25 ± 7.89	4.37 ± 16.64	3.28 ± 13.20	2.19 ± 8.32	2.73 ± 9.22	1.36 ± 10.24	21.2 ± 24.22	18.1 ± 19.86	10.6 ± 15.89	15.0 ± 22.88
Troublesome Coughing	1.11 ± 10.69	8.74 ± 17.11	8.20 ± 15.70	1.64 ± 7.27	1.64 ± 9.48	1.47 ± 9.63	12.1 ± 16.41	19.7 ± 26.55	3.03 ± 9.81	6.67 ± 23.19
Troublesome Talking	0.78 ± 3.64	20.77 ± 30.53	9.29 ± 15.07	1.09 ± 5.98	0.55 ± 4.27	0.55 ± 4.65	18.1 ± 28.60	18.1 ± 24.62	4.55 ± 11.71	1.67 ± 7.45

## Data Availability

Not applicable. The data presented in this study are available on request from the corresponding author. The data are not publicly available due to confidentiality. We thank the patients who volunteered to participate in this study and the staff members who cared for these patients.

## Supplementary Materials

The following are available online at https://www.mdpi.com/1718-7729/28/1/68/s1. Figure S1: The comparison of QLQC-30 between the two groups. Figure S2: The comparison of OES18 between the two groups.
