# Supplementary material for: Comparison of Up-Front Minimally Invasive Esophagectomy versus Open Esophagectomy on Quality of Life for Esophageal Squamous Cell Cancer
Source: Curr Oncol. 2021 Jan 25;28(1):693–701. doi: 10.3390/curroncol28010068 (PMC7924373; doi:10.3390/curroncol28010068)
Supplement: Supplementary file 1 [file curroncol-28-00068-s001.pdf]

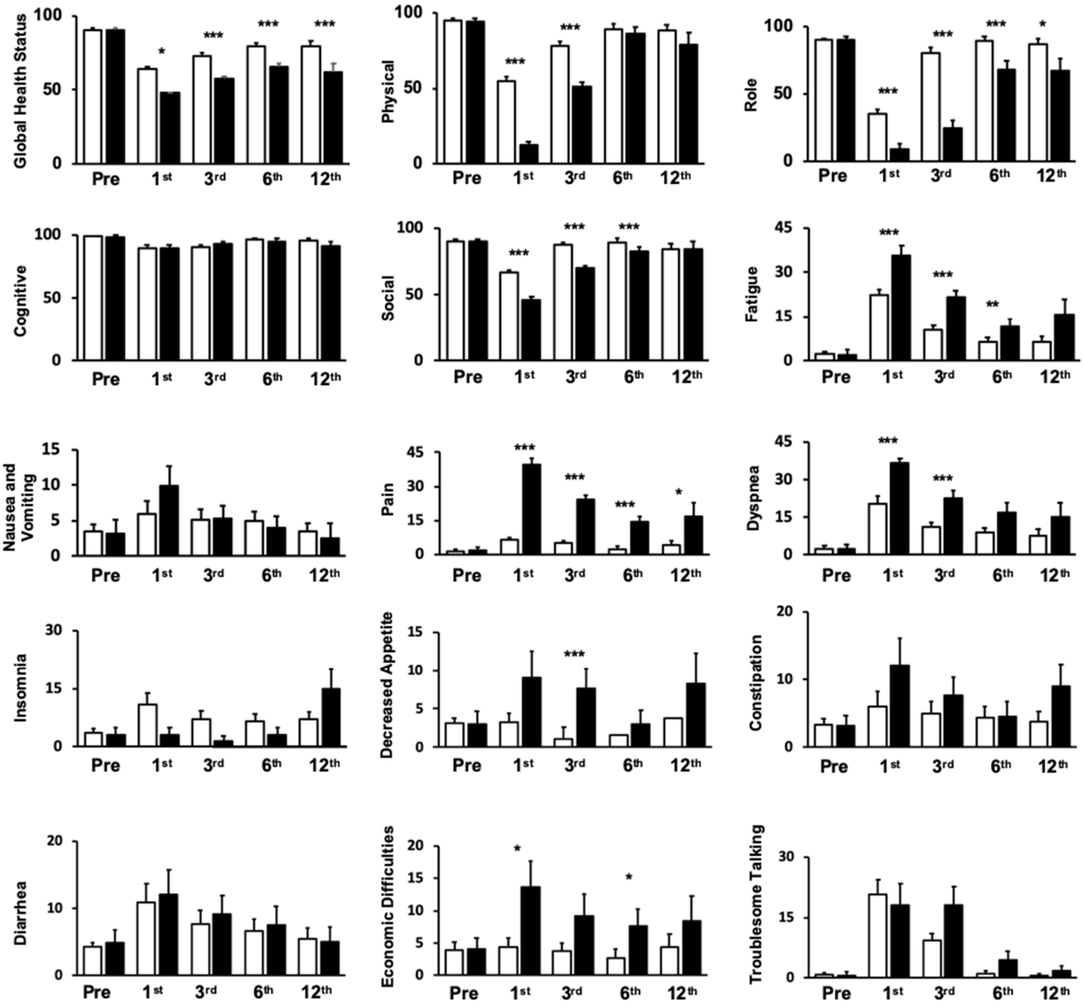

Figure S1. The comparison of QLQC-30 between the two groups.

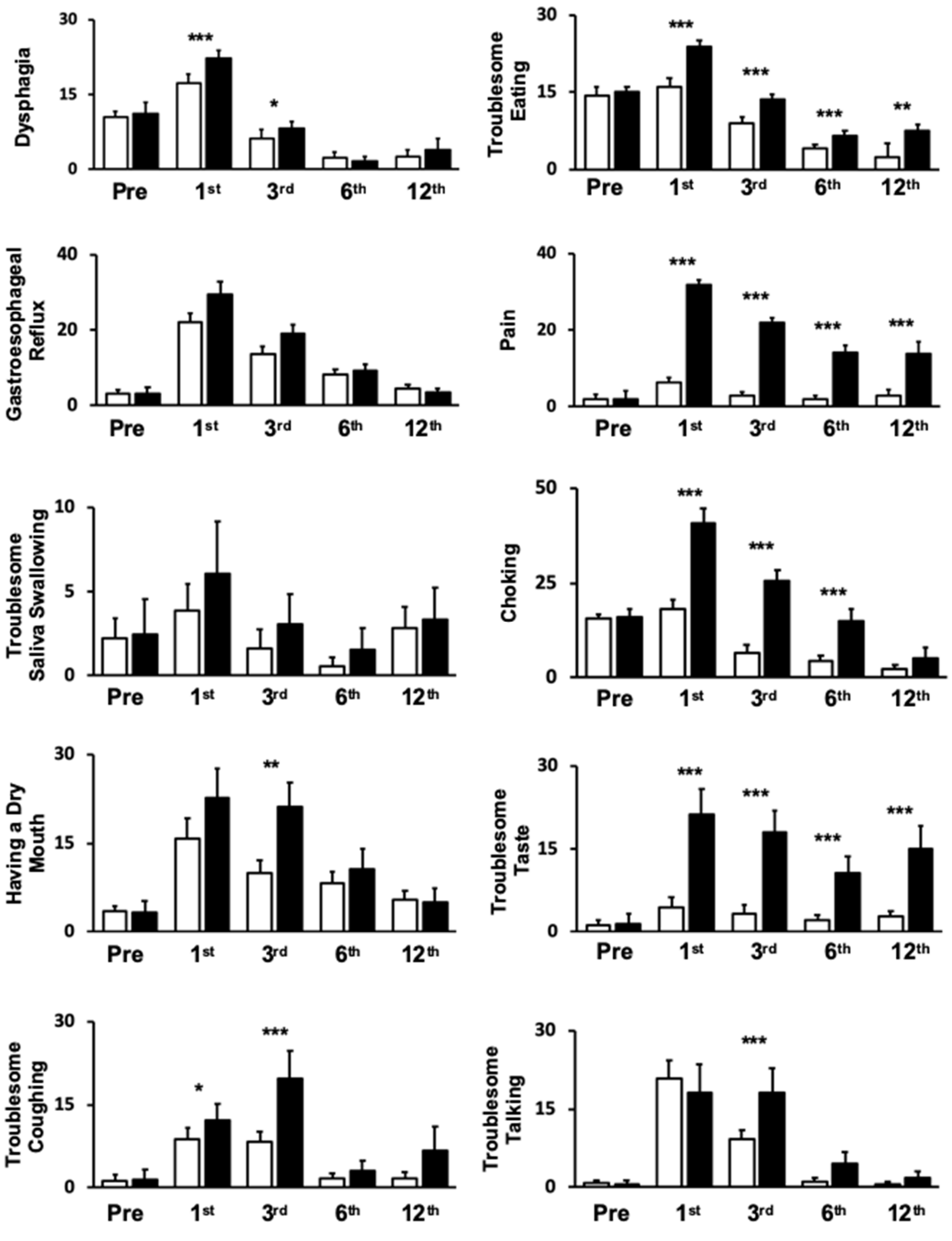

Figure S2. The comparison of OES18 between the two groups.
